# “What a Girl Wants”: What Can We Learn From Animal Models of Female Sexual Motivation?

**DOI:** 10.3389/fnbeh.2019.00216

**Published:** 2019-09-20

**Authors:** Fay A. Guarraci, Russell J. Frohardt

**Affiliations:** ^1^Department of Psychology, Southwestern University, Georgetown, TX, United States; ^2^Academic Success, Northwest Vista College, San Antonio, TX, United States

**Keywords:** paced-mating behavior, partner-preference test, mate choice, solicitation behavior, rats

## Abstract

Sexual motivation is notably different than other motivations such as hunger and thirst, because it lacks homeostatic drive. Sexual motivation poses no threat to physical well-being; individual survival is not at stake. Nevertheless, sexual motivation is a powerful drive and is critical for species survival. Understanding the complexity of sexual motivation has the potential to advance our understanding of other motivations, even pathological motivations, such as those associated with substance abuse. The study of motivation that is unique to females has often been neglected. A number of paradigms have been developed to investigate female sexual motivation beyond measuring only the lordosis reflex. Lordosis is a reflexive posture displayed by female mammals in response to male sexual stimulation to facilitate intromission. The lordosis reflex is essential, but studying the drive to mate is compromised in the absence of robust lordosis. Therefore, appetitive measures of sexual behavior (e.g., preferences, solicitation behaviors) are more specific and more sensitive indicators of sexual motivation than lordosis alone. Paradigms designed to study female sexual motivation often provide a female subject with the choice to interact with a sexually vigorous male or either a non-sexual partner (i.e., female, castrated male) or to remain alone. The study of appetitive measures of sexual motivation has elucidated the role of hormones in female sexual motivation, as well as the underlying neural pathways. The present review describes methods for studying female rats to advance our understanding of sexual motivation and sexual dysfunction.

## Female Sexual Motivation Modeled in Rats

Motivation for sex is unlike many other drives, in that sex lacks a homeostatic drive for balance. Early theories of motivation relied on the assumption that an organism is motivated by an experience of deprivation that creates a need, subsequently activating drives, and then behaviors, which are directed toward a beneficial goal, relieving deprivation (Hull, 1943). Because there is no necessary “deprivation state”, “set point” or “optimal” amount of sex, it is difficult to account for the motivation resulting in sexual behavior with a concept that starts with deprivation. However, in most females – across species – sexual motivation can only be observed when fertilization is possible; if the female is not approaching ovulation, no sexual behavior is displayed and sexual motivation is low. A female rat will avoid a male rat during all phases of her estrous cycle (metestrus, diestrus), except for behavioral estrus (i.e., proestrus). The day of proestrus is characterized by a rise in gonadal hormones (e.g., estrogen followed by progesterone) in anticipation of ovulation. This period of behavioral estrus lasts approximately 24 h. It starts abruptly and ends abruptly (Chu and Agmo, 2015a, b). Therefore, sexual motivation in most mammalian females can only be measured during a limited period of time. During this time, females will display the lordosis reflex. The lordosis reflex is defined as the dorsal flexion of the female rat’s back in response to physical contact (e.g., mounting) from a male rat (Beach, 1976). The lordosis posture facilitates penile penetration and reflects a female’s willingness to receive sexual stimulation from the male (i.e., sexual receptivity). However, because lordosis is a reflex in response to physical contact from the male, it lacks elements of what many consider the basic element of motivation – drive. In many species, including humans, the time-sensitive willingness to engage in sex contributes to the observation that in most species, males have a higher drive for sex than females. Approach behavior is often used as a measure of, and surrogate for, drive. If organisms are motivated to acquire a goal (e.g., food, water, drugs), they will actively seek out and approach the goal. Initially, goal-directed sexual motivation was studied using instrumental conditioning (Everitt and Stacey, 1987; Everitt et al., 1987; Everitt and Wolf, 2002), much like early studies of drug reward. However, these experiments required extensive training and pairing of sexual stimuli with instrumental responses. The present review attempts to identify more parsimonious measures of female sexual motivation. Over the last 40 years, a number of paradigms have been developed to specifically measure female sexual behavior and quantify sexual motivation. The study of female sexual motivation has turned out to be a complicated and nuanced endeavor.

## Laboratory Paradigms That Measure Female Sexual Behavior and Motivation

**FIGURE 1:**
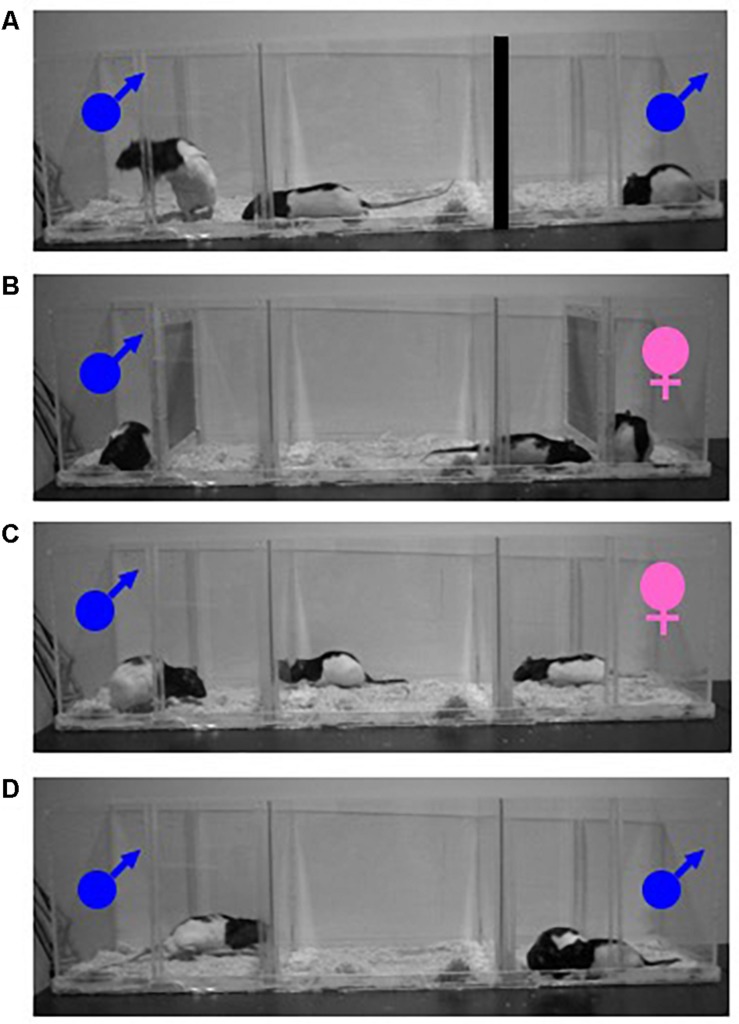
Photograph of a typical paced-mating behavior test where a female rat can mate with one male rat **(A)**. Photograph of a partner-preference test where physical contact is restricted between a female rat (center compartment) and a male stimulus (left compartment) or a female stimulus (right compartment). Both stimulus animals behind wire mesh **(B)**. Photograph of a partner-preference test where physical contact is not restricted between a female rat (center compartment) and a male stimulus (left compartment) or a female stimulus (right compartment) **(C)**. Photograph of a mate choice test where a female rat could interact freely with either of two male stimulus animals **(D)**.

One of the first advances in the study of female sexual motivation involved studying wild and domesticated rats in a semi-natural environment (McClintock and Adler, 1977; McClintock and Anisko, 1982; McClintock et al., 1982). The environment was developed such that female rats could control the rate, or pace, of sexual contact. In the *paced-mating behavior* paradigm, a sexually receptive female is given the opportunity to approach and withdraw from a sexually vigorous male, thereby controlling the timing of mounts, intromissions, and ejaculations (i.e., sexual stimulations). Female rats will pace the receipt of sexual stimulation in semi-naturalistic conditions, as well as in more minimal laboratory settings. This paradigm has been used extensively to model naturalistic aspects of female sexual behavior and quantify female responses (Erskine, 1989; Blaustein and Erskine, 2002). Typically, in this paradigm a female rat is given the opportunity to enter through holes in a divider that separate the subject from a male rat. In Figure 1A, a female rat is depicted leaving the center compartment and approaching the male rat on the left. Access to another male on the right is prevented by blocking the holes in the divider. Furthermore, when female rats can control the rate at which they receive sexual stimulation from one or more males simultaneously, we can assess how the specific measures of paced-mating behavior (e.g., percentage of exits: likelihood of leaving the male after sexual stimulation; contact-return latency: latency to return to the male after sexual stimulation) reflect female sexual motivation. For example, changes in the latency to return to the male after receiving sexual stimulation reflect changes in motivation, with faster return latency indicating an increase in motivation to mate. Differences in percentage of exits are also sensitive to motivational state. For instance, more intense genital stimulation (mount < intromission (mount + penetration) < ejaculation) increases the likelihood of the female’s withdrawal and leads to longer periods away from the male (Erskine, 1989). Allowing the female to pace sexual contact with one or more males is similar to the mating conditions of rats in their natural habitat (Calhoun, 1962). Paced-mating behavior is associated with larger litters (Coopersmith and Erskine, 1994) and is more rewarding (Paredes and Vazquez, 1999; Martinez and Paredes, 2001) for the female, when compared to non-paced conditions. The observation of paced-mating behavior in females, makes understanding female sexual motivation more complicated than male sexual motivation, given that there are aspects of a sexual encounter that seem to drive females *away* from males in the middle of a sexual encounter. The complexity of female sexual behavior is even more problematic when viewing this behavior through a lens common to the study of motivation; more approach = more motivation. Female sexual behavior is not endless approach behavior, suggesting that not all aspects of sexual contact are equally motivating. Therefore, sexual behavior in the female rat becomes a delicate balance between approaching the male and avoiding the male (Paredes and Vazquez, 1999). Somatosensory stimulation received from the male, and female motivation, act in concert to affect female behavior and likely contribute to the avoidance of the male during mating (Erskine et al., 2004; Clark et al., 2011). Because receipt of sexual stimulation triggers withdrawal, the amount of time spent with the male is reduced when mating is possible, relative to when the female can only exchange olfactory, visual, and auditory stimuli, but not mate with the male (Clark et al., 2004). The control of the timing of sexual contact is not only rewarding for females (Paredes and Vazquez, 1999; Martinez and Paredes, 2001), but also increases fertility. Therefore, the somatosensory stimulation experienced during intromission and insemination (Komisaruk and Wallman, 1977) may have been essential for the development of paced mating in the species and contributes to the rewarding qualities of vaginocervical stimulation.

The *partner-preference test* is a paradigm commonly used to evaluate approach and the appetitive aspects of sexual behavior (Paredes and Alonso, 1997; Avitsur and Yirmiya, 1999; Paredes and Vazquez, 1999; Bakker, 2003). During a partner-preference test, a sexually receptive female is given the choice to spend time in the vicinity of either a sexual partner (e.g., sexually vigorous male) or a non-sexual partner (e.g., same-sex conspecific, castrated male). A sexually receptive female rat will spend more time with the sexual partner when the sexual partner is placed behind a wire mesh thereby restricting physical contact (Figure 1B), than when physical contact is not restricted and mating is possible (Figure 1C). The difference between preferences observed when physical contact is restricted vs. when physical contact is unrestricted indicates that the distal cues (i.e., auditory, visual and olfactory) of a sexual partner are not only sufficient for approach behavior but these cues elicit a more robust preference in female rats (Clark et al., 2004). Because female rats spend less time with a sexual partner under conditions that also allow them to engage in paced-mating behavior, it is possible that some aspects of sexual stimulation received during paced mating may be aversive to female rats. Alternatively, the difference between the two conditions of the partner-preference test could also be a function of the very nature of paced-mating behavior. Specifically, leaving the male after the receipt of sexual stimulation followed by periods of time remaining away from the male could artificially reduce the time that a female rat can spend with a sexual partner.

The *conditioned-place preference (CPP)* paradigm has also been used to assess the rewarding aspects of sex. Although the CPP paradigm has been useful in assessing the rewarding properties of drugs that are commonly abused, such as opiates and psychomotor stimulants (Carlezon, 2003), it has also been used to identify which aspects of sex and under which conditions do female rats find sex rewarding. In the CPP paradigm, aspects of a sexual encounter (e.g., conditions for mating, types of mating stimulation) are repeatedly paired with spending time in one distinct context (e.g., white walls, gravel floor), whereas another distinct context (e.g., black walls, grate floor) is paired with a control condition (e.g., no mating). If aspects of a sexual encounter were sufficiently rewarding, an association between the context and sexual encounter will develop. Evidence of this reward state will be expressed by subjects as a preference to spend time in that conditioned context when given the opportunity to spend time in either context. Initial studies found that paced-mating behavior could be conditioned, therefore female control over the timing of mating is rewarding (Paredes and Alonso, 1997). Furthermore, pre-treatment with naloxone (i.e., opiate antagonist) blocks the formation of a CPP associated with female paced sexual stimulation, indicating that the rewarding properties of paced-mating behavior depend on opioid receptors (Paredes and Martinez, 2001). However, a number of studies have since suggested that what is rewarding is not necessarily control *per se*, but allowing the female to take a break between sexual stimulation. For example, Becker and colleagues reported increases in mesencephalic (i.e., striatum, nucleus accumbens) dopamine release in response to copulation if the female experiences her “preferred pacing interval” between sexual stimulations, even when the female had no active control of this interval (Jenkins and Becker, 2001, 2003a,b). Meerts and Clark (2007, 2009) have also found that vaginocervical stimulation (VCS) is rewarding when measured using the CPP paradigm, independent of active control (i.e., artificial VCS or non-paced mating conditions), as long as females are given a brief period of time without any sexual stimulation following ejaculations (the most intense sexual contact), suggesting that the reprieve from sexual stimulation is critical for the reward state.

The *mate choice* paradigm is another methodology that has been used to advance our understanding of the rewarding properties of sex in female rats. Although rats are promiscuous, preference for one mate over another has been observed. In this paradigm, female rats are given the choice to mate with multiple male rats simultaneously (Figure 1D). Choice of one mate over another can be determined by which mate the female spends more time with and/or which mate is visited first. Results from our lab have consistently found that a female rat will spend more than twice as much time with one mate (i.e., her preferred mate) than another (i.e., her non-preferred mate), as well as return faster to her preferred mate than to her non-preferred mate following sexual stimulation. In addition, female rats receive more sexual stimulations from their preferred mate than their non-preferred mate. Female rats will visit and display solicitation behaviors more frequently with their preferred mate than their non-preferred mate (Ferreira-Nuño et al., 2005; Lovell et al., 2007; Zewail-Foote et al., 2009). The pattern of behavior displayed with a preferred mate further supports the conclusion that measures of paced-mating behavior reflect sexual motivation. Specifically, females are less likely to leave their preferred mate than their non-preferred mate after receiving sexual stimulation, but if they do leave, they return to their preferred mate faster than their non-preferred mate. In addition to describing the patterns of mate choice in female rats, we have also investigated the effects of mate choice on reproductive success (Lovell et al., 2007; Zewail-Foote et al., 2009). From these studies, we have found that female rats consistently prefer the same mate across multiple tests, as well as between different females. However, using the mate choice paradigm or olfactory preferences for particular mates, we have been able to determine that it is unlikely that preference for a particular male rat is related to urinary testosterone levels, body weight, or testes weight (Winland et al., 2012) but instead female rats are attracted to males with high levels of major urinary proteins, which could communicate health, nutritional status, and social rank (Kumar et al., 2014). Surprisingly, mate choice does not seem to provide any reproductive advantage (Winland et al., 2012; Chu et al., 2015).

During any mating encounter, in any paradigm, female rats also display species-specific, sex-specific behaviors, such as hopping, darting, ear wiggling, and presenting (Erskine, 1989). These additional behaviors displayed by sexually receptive female rats seem to attract or “solicit” the attention of potential mates, hence the common use of the term “solicitation behaviors” to describe this cluster of behaviors. Although the underlying neural mechanism of these behaviors is not well known, solicitation behaviors often precede the receipt of sexual stimulation from a male, suggesting that there is a functional purpose for these behaviors. Not all female rats display solicitation behaviors consistently throughout a mating encounter, nevertheless, interest in sexual contact has been inferred from the rate at which females attract a male rat’s attention with the display of hops, darts, ear wiggles, and presentations. New qualitative analyses of complex sequences of behavior may be useful in furthering our understanding of the role solicitation behaviors play in a sexual interaction.

## Indications of Motivation

A consistent pattern of behavior has been identified in the many recent experiments investigating female sexual behavior using the aforementioned paradigms. For example, following the administration of a number of different psychomotor stimulants that are known to enhance, or cross-sensitize with, other reinforcing drugs, female rats have been shown to spend more time with a sexual partner, leave a sexual partner less frequently, and display more solicitation behaviors (Guarraci, 2010; Guarraci and Bolton, 2014). This pattern of behavior likely reflects an increase in a female rat’s motivation to spend time interacting with a male rat. Alterations in motivation do not always follow the above pattern perfectly; rarely do we observe females spending more time, leaving less often, coming back faster, and displaying more solicitations as the result of a drug treatment or other experimental manipulation. More often than not, we see the pattern with two or three of these behaviors affected. For instance, ketamine, at doses comparable to what is being used off-label to treat depression, increased time spent with a male rat during a partner-preference test and decreased the likelihood of leaving the male after sexual stimulation (Guarraci et al., 2018). In addition to pharmacological studies, we have also observed this pattern of enhanced motivation to mate under other conditions. The pattern is observed while a female is mating with her preferred mate (as mentioned above). This pattern is also observed after repeated mating encounters, as female rats transition from virgins to experienced breeders. With regular repeated sexual experience, female rats spend more time with a male, are less likely to leave after sexual stimulation, return to the male faster, and display more solicitation behaviors, when compared to virgin females during their first sexual encounter (Meerts et al., 2014, 2016; Guarraci and Meerts, 2017; Arnold et al., 2019; Piergies et al., 2019).

In contrast, a variety of conditions result in a consistent pattern indicating a disruption of sexual motivation beyond the lordosis reflex. This pattern is characterized by female rats spending less time with a sexual partner, leaving the male more frequently, taking longer to return after receiving sexual stimulation, and displaying fewer solicitation behaviors. We have observed this disruptive pattern when female rats are exposed to drugs that block estrogen receptors or drugs that inhibit PDE-5 (Clark et al., 2003, 2009). Lesions of the medial preoptic area of the hypothalamus also decrease time spent with a sexual partner, increase the likelihood of leaving, and delay returning to the male after receiving sexual stimulation (Yang and Clemens, 2000; Guarraci et al., 2004; Guarraci and Clark, 2006) when subjects are tested for partner preference and during paced-mating behavior. Interestingly, this pattern was observed even though the lordosis reflex remained intact; motivation of the female rat to actively pursue sexual contact was diminished by the lesions despite robust lordosis.

An important consideration must be made when a manipulation or treatment changes levels of general locomotor behavior. For example, psychomotor stimulants increase locomotion. Such increases in locomotion can artifactually affect measures we record during mating tests, such as visits to the stimulus animals. In contrast, opiates and aging have been shown to decrease general locomotor behavior. In lieu of these changes in locomotion, we have had to rely on discrimination between the male and the female stimulus. Specifically, even when locomotor behavior is increased following administration with caffeine, we have noted that visits to the male stimulus outpace visits to the female, indicating discriminating motivation (Guarraci and Benson, 2005). Similarly, we have noted increases to the male stimulus compared to the female stimulus despite overall decreases in visits to both stimulus animals in middle-aged female rats (under review).

Taken together, the growing literature investigating female sexual motivation indicates that sexual behavior in the female rat a complex balance between approach and withdrawal that can be measured with a number of paradigms. Studies of sexual motivation in female rats can be used to advance our understanding of the underlying neural pathways of healthy motivation, as well as dysfunctional motivation.

## Author Contributions

Both authors worked equally on the development of the ideas and the writing.

## Conflict of Interest Statement

The authors declare that the research was conducted in the absence of any commercial or financial relationships that could be construed as a potential conflict of interest.
